# Developing Odontoma with Radiolucency in the Maxillary Right Second Molar

**DOI:** 10.3390/reports9030238

**Published:** 2026-07-22

**Authors:** Tatsuya Akitomo, Yuria Asao, Nanako Kataoka, Toshinori Ando, Mikihito Kajiya, Ryota Nomura

**Affiliations:** 1 Department of Pediatric Dentistry, Graduate School of Biomedical and Health Sciences, Hiroshima University, Hiroshima 734-8553, Japan; 2 Department of Oral and Maxillofacial Pathobiology, Graduate School of Biomedical and Health Sciences, Hiroshima University, Hiroshima 734-8553, Japan; 3 Center of Oral Clinical Examination, Hiroshima University Hospital, Hiroshima 734-8551, Japan

**Keywords:** case report, odontoma, pediatric dentistry

## Abstract

Odontoma is one of the most frequent odontogenic tumors, and most cases occur in pediatric patients. A panoramic examination of an 11-year-old boy presenting with a chief complaint of dental caries revealed delayed eruption of the maxillary right second molar and a radiolucency around the crown. In addition, cone-beam computed tomography revealed slight calcifications within a cystic lesion measuring 20 × 18 × 13 mm, and demonstrated root resorption of the first molar. Following root canal treatment of the first molar, the lesion was removed under general anesthesia. Histological findings of the resected lesion confirmed the diagnosis of a developing odontoma. This report highlights the importance of early detection of odontomas through radiographic examination.

Odontogenic tumors constitute approximately 3% of oral lesions, among which odontoma is the most frequent, accounting for 22% [1]. The reported prevalence of odontoma ranges from 0.24% to 1.21%, and although data vary among studies, pooled analyses suggest that the male-to-female ratio is nearly 1:1 [2,3]. It is common in pediatric patients, with some reports indicating that individuals younger than 18 years account for approximately 70% of cases [4]. Odontoma may cause a range of complications and can interfere with tooth eruption; therefore, early detection and management by a pediatric dentist are important [3]. Odontoma are classified into compound and complex types, and although the internal architecture of the calcified component differs between the two types, both are surrounded by a narrow radiolucent area [5]. In the present case, the patient was referred to our hospital for caries treatment. Panoramic examination for dental caries revealed the lesion (Figure 1A–C) and histological examination ultimately confirmed the diagnosis of developing odontoma. Although there are many reports regarding odontomas developed within cysts, there are few reports about developing odontomas [6,7]. At first, an enlarged radiolucency around the crown of the maxillary right second molar was detected, and suspected cysts of the jaws or odontogenic tumors. In the 5th edition of the World Health Organization Classification of Head and Neck, cysts of the jaws include dentigerous cyst and radicular cyst [8]. On the other hand, odontogenic tumors include calcifying epithelial odontogenic tumor, ameloblastic fibroma, and odontoma [8]. Although most benign odontogenic tumors are radiolucent, odontomas are characterized by a radio-opaque appearance [9]. In the present case, a dentigerous cyst was initially suspected based on a well-circumscribed, unilocular radiolucency surrounding the crown of the impacted tooth [10]. CBCT revealed the full extent of the cyst, internal calcifications, and root resorption of the adjacent tooth (Figure 2, Figure 3 and Figure 4). The presence of internal calcifications was a finding that supported the diagnosis of an odontogenic tumor. However, both dentigerous cyst and odontogenic tumors can cause root resorption; therefore, a definitive diagnosis could not be established at the time of the CBCT examination [11,12]. In addition, because both cysts of the jaw and odontogenic tumors exhibit a variety of characteristic histological findings, pathological examination was crucial for diagnosis. Costa et al. (2015) reported a pediatric patient with a complex odontoma associated with an odontogenic cyst [6]. In that case, panoramic radiography demonstrated a well-defined radiolucent lesion containing radiopaque material, whereas in the present case, calcifications were detected only on CBCT [6]. Although rare, this indicates that a minute odontoma, too small to be detected on a panoramic radiograph, may be present within a cyst-like lesion and identified only on CBCT.

Histopathological examination of the resected specimen revealed scattered islands of odontogenic epithelium embedded within a pale basophilic dental papilla-like stroma containing myxoid components (Figure 5A–D). Multiple nodular proliferative foci composed of relatively cellular spindle-shaped cells were observed. In some areas, these nodules were continuous with the odontogenic epithelial component and were associated with the formation of eosinophilic hyalinized material considered to represent osteodentin matrix. Based on these histopathological findings, the lesion was diagnosed as a developing odontoma. A dentigerous cyst was excluded because the lesion lacked a lining of non-keratinized stratified squamous epithelium and exhibited evident hard tissue formation. In addition, other odontogenic tumors associated with hard tissue formation were ruled out based on their characteristic histopathological features. In this case as well, pathological examination achieved a definitive diagnosis of odontoma. Even if there is only a small amount of calcification within a cystic lesion, it may be an odontoma. This report highlights the importance of suspicion of odontoma even in cystic lesions.

Additional procedures such as apicoectomy, extraction, and preoperative root canal therapy lower the risk of complications in patients undergoing cyst enucleation of the jaw [13]. However, because the tooth’s vitality is lost, preoperative root canal therapy must be carefully considered, weighing the possibility of regeneration against the risk of complications [13]. Electric pulp test is useful for determining preoperative canal treatment of adjacent teeth to cysts in anterior teeth [13]. In the present case, radiological examination did not rule out the possibility of a tumor. Therefore, considering the risk of recurrence, it was decided to perform preoperative root canal treatment (Figure 6A–C). An electric pulp test was not performed because the tooth had deep caries and a pre-existing restoration, and accurate evaluation would likely not have been achievable.

The patient was diagnosed with an intellectual disability, and the caries treatment was performed under restraint. However, the patient’s cooperation with dental treatment improved during the course of caries treatment, and restraint was not required during root canal treatment. Intravenous sedation is a safe anesthetic method, similar to general anesthesia, and it is also performed in oral surgery [14]. On the other hand, sedation in pediatric patient may not be appropriate for some procedures, such as long procedures or invasive procedures [15]. In the present case, considering these factors, the oral surgeon chose to treat the patient under general anesthesia. Follow-up research reported that 11.5% of odontogenic tumors cases exhibit recurrence, and long-term follow-up is important [16]. After surgical treatment, we continued the follow-up, including radiographic examinations such as panoramic and periapical radiographs. Although a radiolucency was detected at the apex of the second molar, there was neither a change in the image nor a recurrence of odontoma (Figure 7A–F). Table 1 outlines the timeline of the patient’s diagnosis, treatment, and follow-up.

Most odontomas are asymptomatic and are discovered incidentally during routine radiographic examinations, often in association with eruption disturbances [17]. In addition, eruption disturbances can also occur due to supernumerary teeth or tooth transposition [18,19]. In this case, the patient had not undergone regular dental checkups, and the lesion was identified during panoramic imaging performed for caries treatment. If it had been detected later, the first molar, which was already showing signs of root resorption, might not have been salvageable. When dental professionals identify eruption disturbances, it is important to conduct a detailed examination to determine the cause, and provide appropriate treatment. This report highlights the importance of regular dental checkup including radiographic examination for early detection of eruption disturbances. In addition, even subtle calcified components detectable only on CBCT should prompt consideration of the presence of developing odontoma.

## Figures and Tables

**Figure 1:**
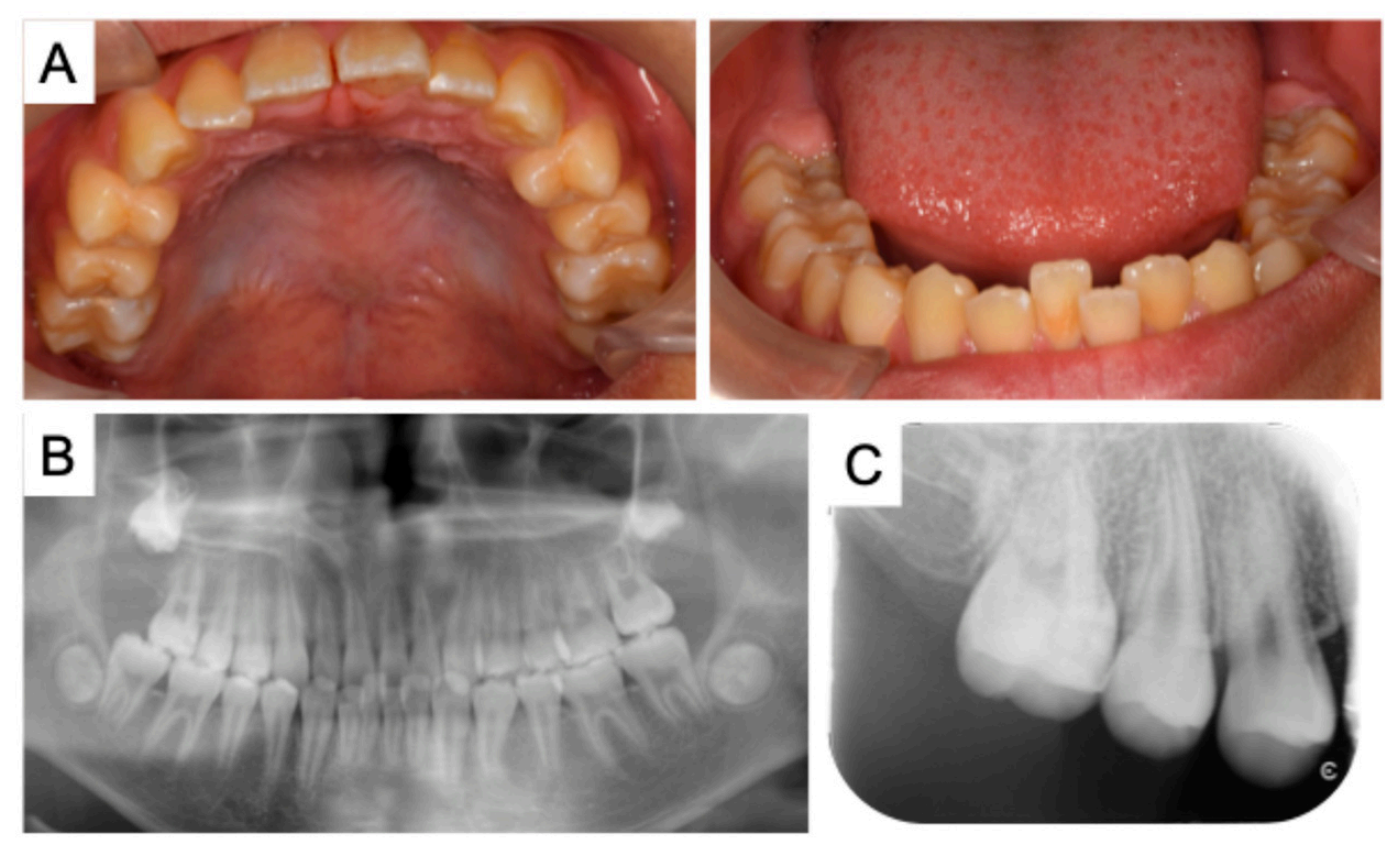
Intraoral and panoramic examinations at the initial visit. An 11-years-and-11-months-old boy was referred to our hospital with a chief complaint of dental caries. A routine school dental checkup had detected the caries. He visited a private dental clinic but was unable to receive treatment because of uncooperative behavior and a pronounced gag reflex. The medical and family histories were otherwise unremarkable, except for intellectual disability. Twenty-seven permanent teeth, except for the maxillary right second molar, had erupted into the oral cavity (**A**). Panoramic examination revealed the presence of the maxillary right second molar, with a well-defined radiolucency surrounding the crown (**B**). Root formation was slightly delayed compared with the contralateral tooth; however, eruption appeared markedly delayed. A periapical radiograph could not provide adequate evaluation because of the gag reflex (**C**). Dental caries were identified in multiple molars, and caries treatment was prioritized. A detailed examination of the maxillary second molars was scheduled subsequently. Caries treatment was performed under physical restraint with parental consent.

**Figure 2:**
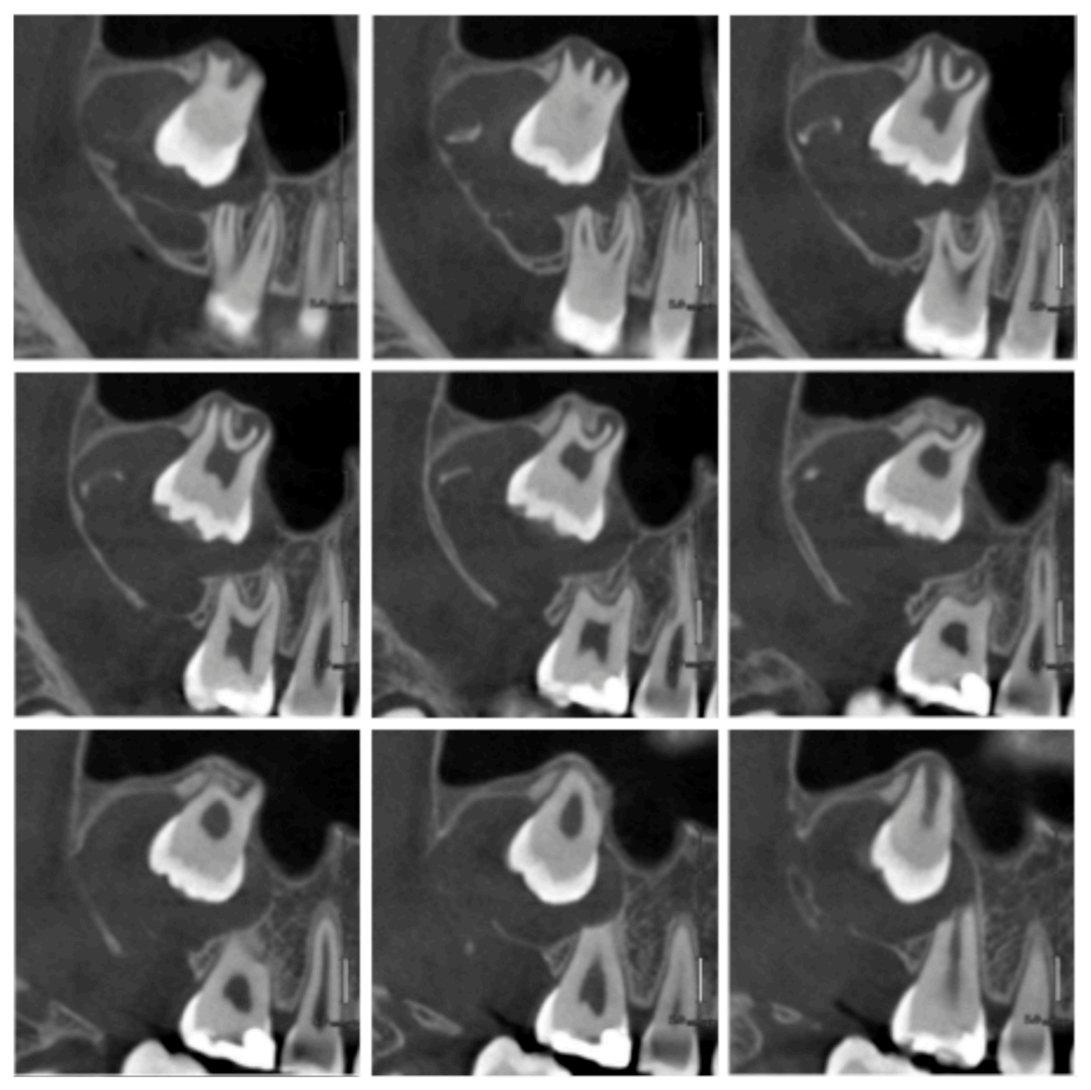
Sagittal section of cone-beam computed tomography (CBCT) images at 12 years 6 months of age, Scale bar = 5 mm. The scanning protocol involved a field of view of 6 cm, voxel size of 125 μm, slice thickness of 0.5 mm, and 8 mA/90 kVp. When CBCT was taken after caries treatment, a radiolucent area measuring 20 × 18 × 13 mm was observed around the crown of the maxillary second molar, with a calcified structure located superior to the second molar. Another calcified structure, resembling a third molar, was also noted in the distal portion of the lesion.

**Figure 3:**
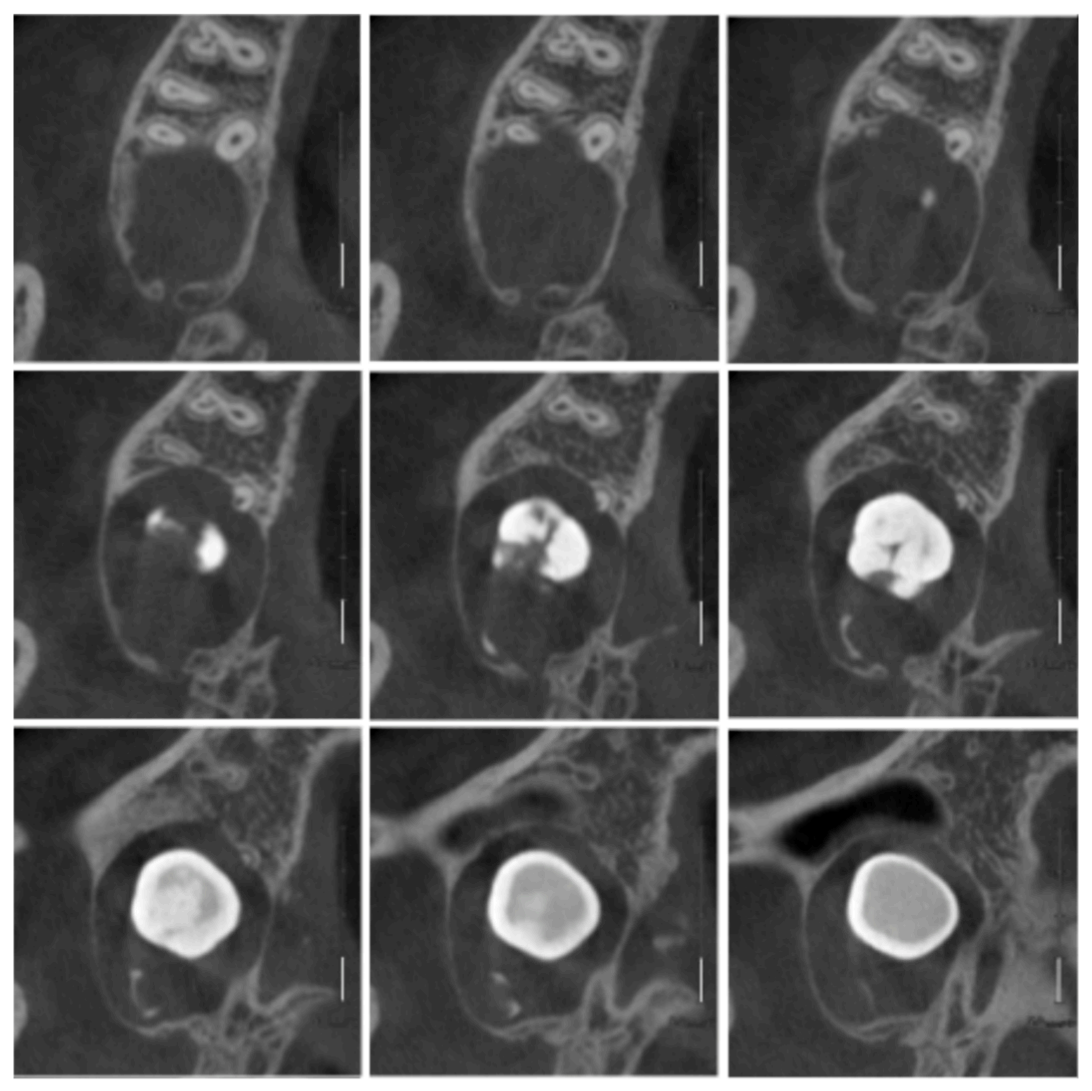
Axial section of CBCT images at 12 years 6 months of age. Scale bar = 5 mm. This revealed the buccal distal root and palatal root were connected to the lesion and exhibited resorption. In addition, cortical bone was thinned and partial disruption was observed.

**Figure 4:**
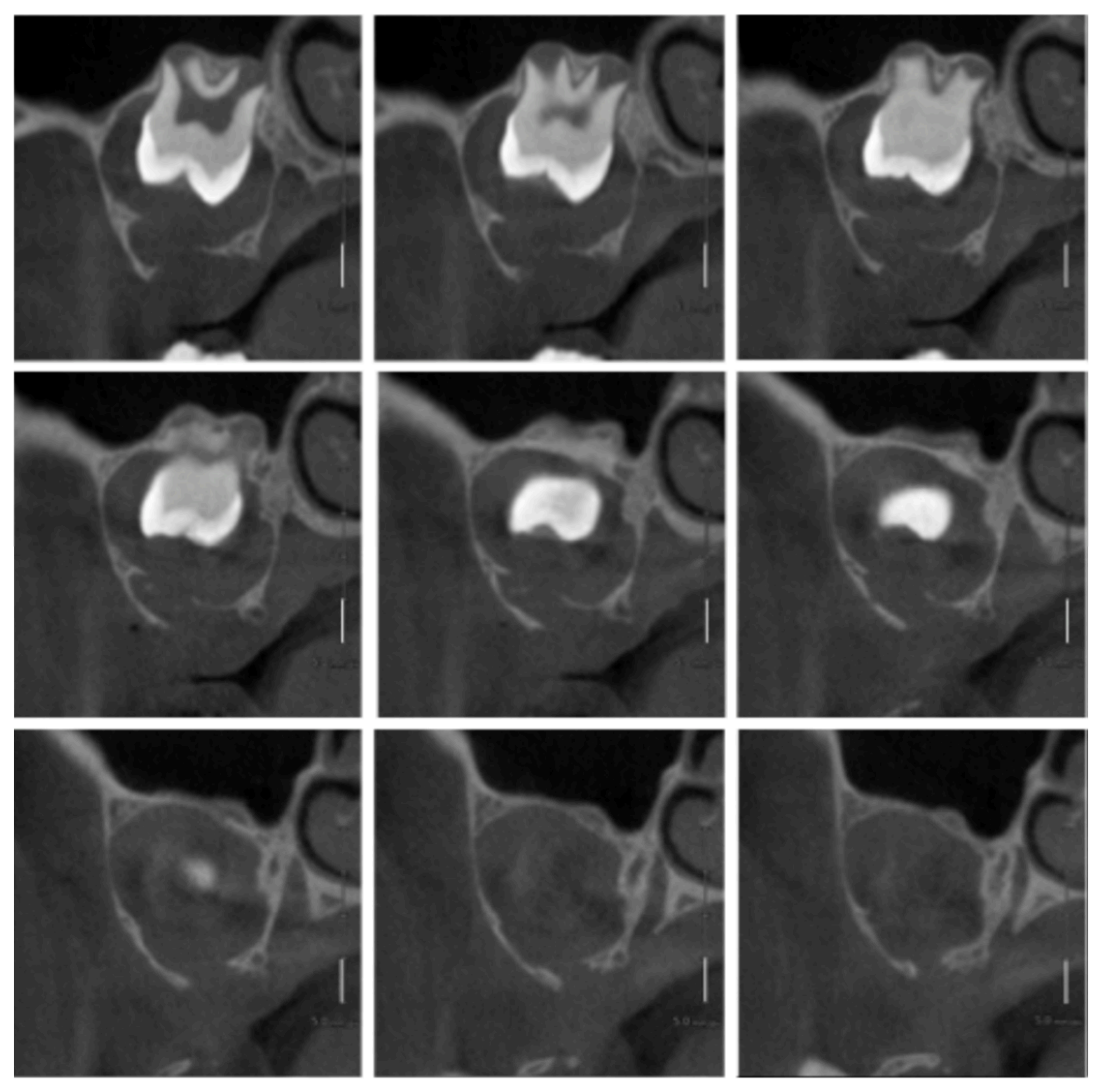
Coronal section of CBCT images at 12 years 6 months of age. Scale bar = 5 mm. Although the second molar was close to the maxillary sinus, a bone of at least approximately 0.5 mm was present. There was no invasion observed into the maxillary sinus.

**Figure 5:**
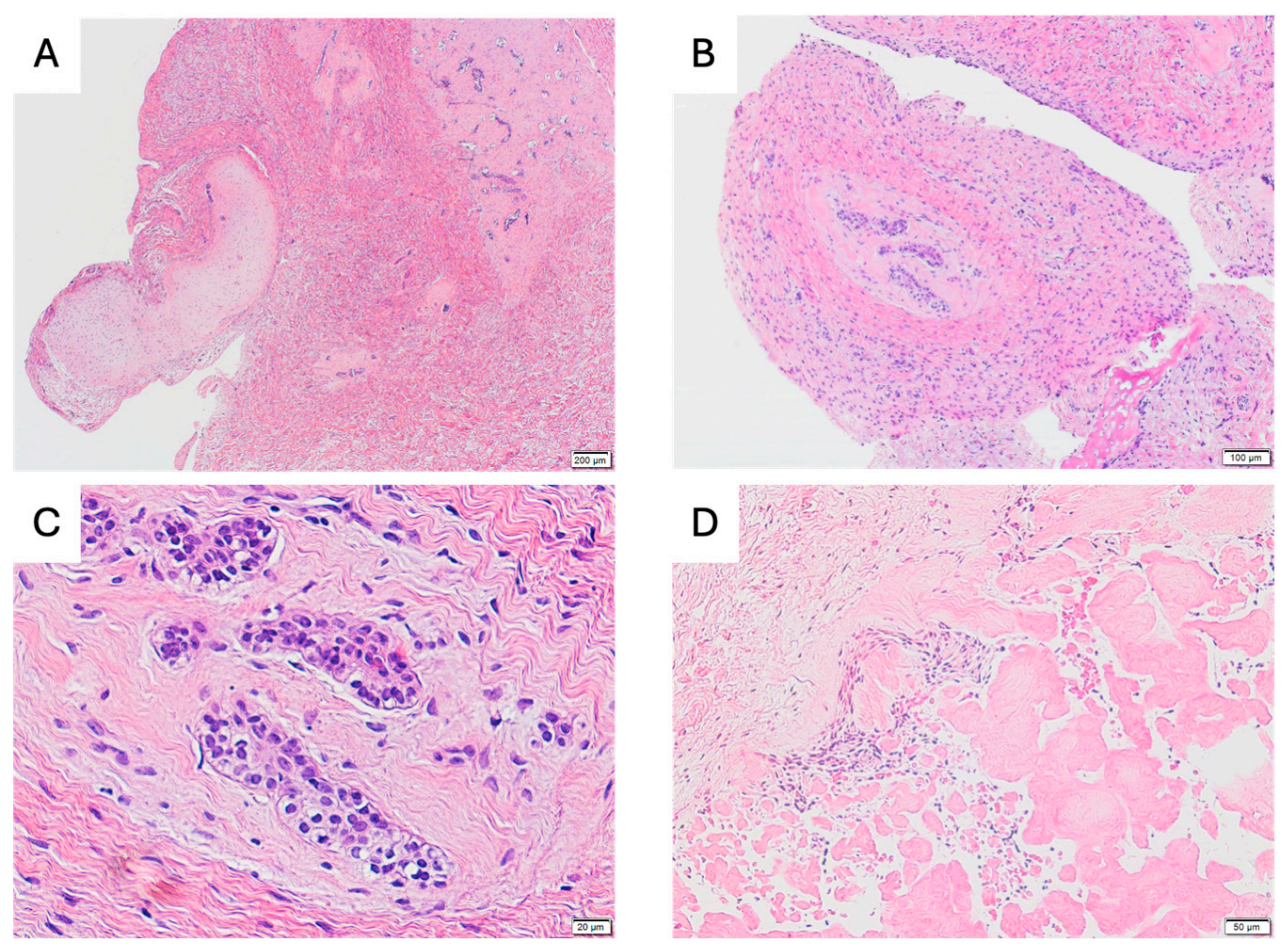
Histological image of removed cystic lesion. Multinodular structures are present within the fibrous connective tissue ((**A**) scale bar = 200 µm). Nodules containing odontogenic epithelial islands are observed ((**B**) scale bar = 100 µm). A partially palisading arrangement of columnar cells is observed in the epithelial islands. The islands are surrounded by a dental papilla-like stroma. ((**C**) Scale bar = 20 µm.) Formation of eosinophilic hyaline material, regarded as dentinoid/osteodentin-like tissue, was also observed ((**D**) scale bar = 50 µm). Based on these findings, the diagnosis of a developing odontoma was confirmed. The wound healed well, and the maxillary first molar was restored with a preformed stainless steel crown 8 months after surgery.

**Figure 6:**
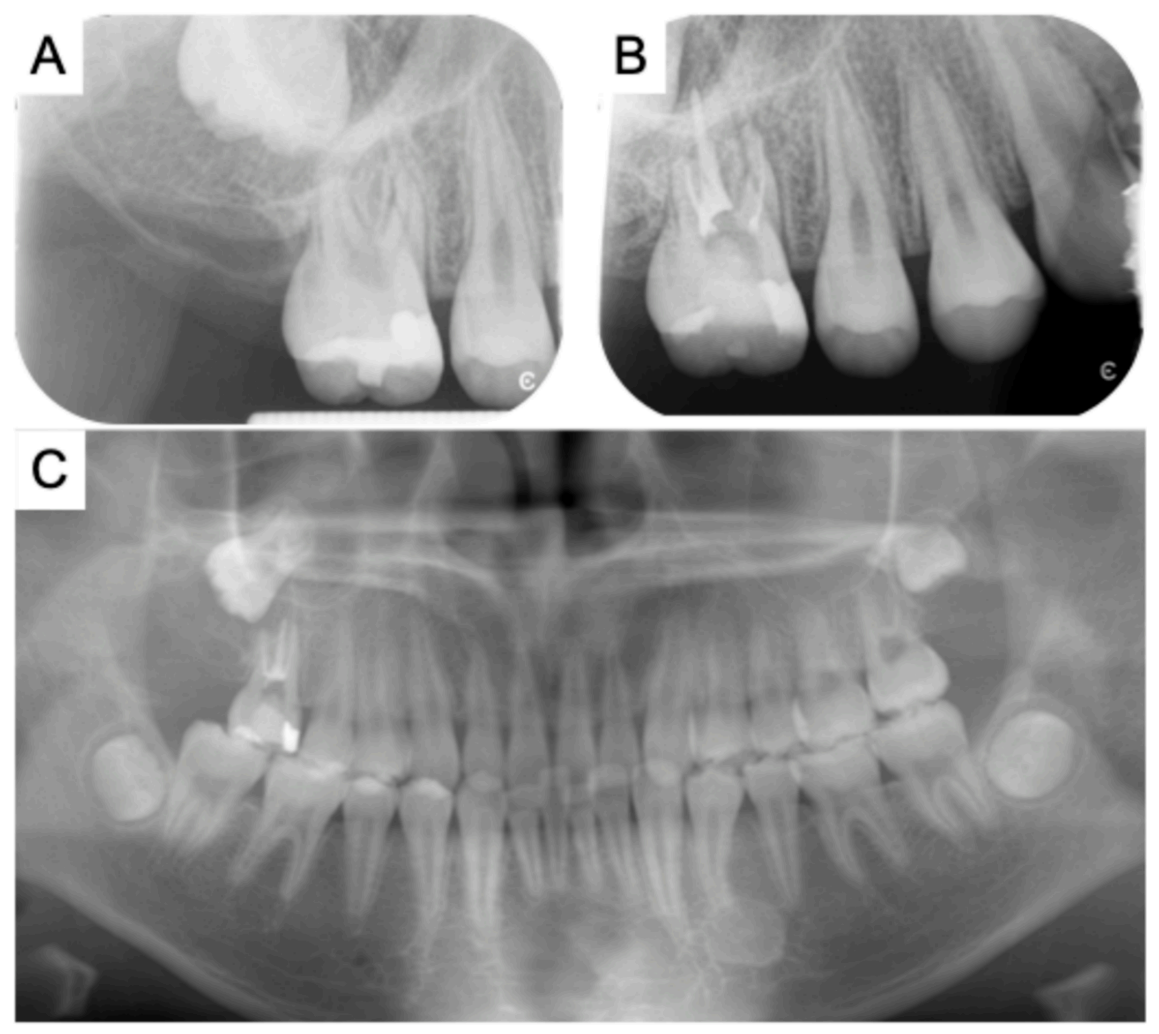
Radiographic examination before and after root canal treatment of the first molar. Based on the clinical diagnosis of a maxillary cyst, provisional pulpectomy of the first molar was initially performed in cooperation with the Department of Periodontology (**A**,**B**). By this time, the patient’s cooperation had improved, and physical restraint was no longer required. In addition, panoramic examination at 13 years and 2 months of age showed no significant changes in the cyst (**C**). On the following day, cystectomy and removal of the impacted second and third molars were performed under general anesthesia in the Department of Oral Surgery. An incision was made in the buccal gingiva in the region of the second premolar and first molar, and the mucoperiosteal flap was elevated. A bone defect was observed; the surrounding bone was removed, and the lesion was extracted en bloc with the second molar. The adjacent third molar was subsequently extracted, and the site was curetted and sutured.

**Figure 7:**
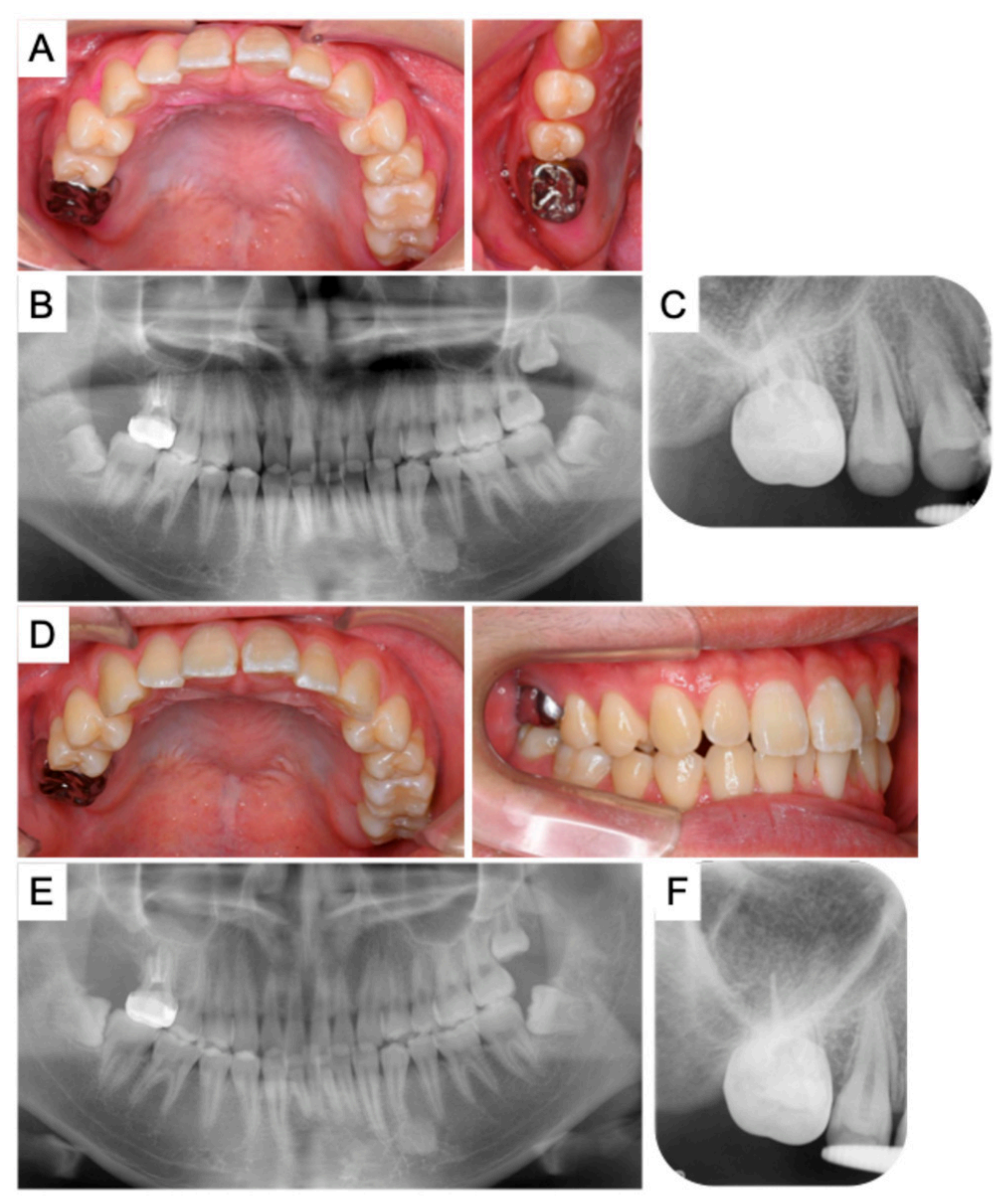
Intraoral and radiographic findings during follow-up. Follow-up was continued, and radiographic examinations were performed at 15 years and 5 months of age. These showed a radiolucency at the apex of the second molar, raising suspicion of apical periodontitis; however, the patient was placed under observation because no other pathological findings were present (**A**–**C**). At 16 years and 8 months of age, the radiolucency remained unchanged, and no recurrence of odontoma was observed (**D**–**F**). In addition, no changes were noted in the bone sclerosis of the mandibular left first premolar, and observation was continued. Oral management was maintained until 17 years 7 months of age, and no retreatment of the maxillary right second molar was required. At the time of this writing, the patient was continuing oral management in the Department of Special Care Dentistry.

**Table 1:** Timeline of the patient’s diagnosis, treatment, and follow-up.

Age	Occurrences
11 years and 11 months	First visit to hospital, and detection of delayed eruption of second molar
12 years and 6 months	Detection of slight calcification within the cystic lesion
12 years and 8 months	Root canal treatment of the first molar
13 years and 2 months	Cystectomy and removal of the impacted second and third molars
13 years and 10 months	Restoration of first molar with a preformed stainless steel crown
15 years and 5 months	Follow-up with radiographic examination
16 years and 8 months	Follow-up with radiographic examination
17 years and 7 months	Final visit to our department and referral to the department of Special Care Dentistry.

## Data Availability

The original contributions presented in this study are included in the article. Further inquiries can be directed to the corresponding author.
